# Improvement of rice blast resistance by developing monogenic lines, two-gene pyramids and three-gene pyramid through MAS

**DOI:** 10.1186/s12284-019-0336-4

**Published:** 2019-11-04

**Authors:** Wuming Xiao, Qiyun Yang, Ming Huang, Tao Guo, Yongzhu Liu, Jiafeng Wang, Guili Yang, Jiyong Zhou, Jianyuan Yang, Xiaoyuan Zhu, Zhiqiang Chen, Hui Wang

**Affiliations:** 1 National Engineering Research Center of Plant Space Breeding, South China Agricultural University, Guangzhou, 510642 People’s Republic of China; 2 Plant Protection Research Institute, Guangdong Academy of Agricultural Sciences/Guangdong Provincial Key Laboratory of High Technology for Plant Protection, Guangzhou, 510640 People’s Republic of China; 3 Guangdong Agricultural Technology Extension Station, Guangzhou, 510520 People’s Republic of China

**Keywords:** Rice, Blast resistance, MAS, Monogenic lines, Two-gene pyramids, Three-gene pyramid

## Abstract

**Background:**

Rice blast caused by *Magnaporthe oryzae* (*M. oryzae*) is one of the most destructive diseases in rice production. Development of resistant varieties through pyramiding of resistant (*R*) genes is considered as an effective strategy to cope with the disease. However, is it really essential to pyramid more *R* genes in a specific ecological regions? To answer this question, a set of rice improved lines were developed in this study. Afterwards, the blast disease resistance and agronomic traits of the recurrent parent (RP), donor parents (DPs) and improved lines were investigated.

**Results:**

We developed seven improved lines, comprising three monogenic lines, three two-gene pyramids and one three-gene pyramid, by introgression of *R* gene(s) into a common genetic background using marker-assisted backcross breeding (MABB). Based on 302 SSR markers, the recurrent genome of the seven improved lines reached a range of 89.1 to 95.5%, with the average genome recovery of 92.9%. The pathogenicity assays inoculated with 32 different blast isolates under artificial conditions showed that the resistance spectrum of all the improved lines was significantly broadened. The assays further showed that the two-gene pyramids and the three-gene pyramid exhibited wider resistance spectrum than the monogenic lines. At natural nurseries, the three monogenic lines still showed high ratios of infected panicles, whereas the two-gene pyramids and the three-gene pyramid showed high level of panicle blast resistance. However, the two-gene pyramid R504 reached the similar resistance effect of the three-gene pyramid R507 considering resistance spectrum under artificial conditions and panicle blast resistance under field conditions. Generally, the improved lines showed comparable agronomic traits compared with the recurrent parent (RP), but the three-gene pyramid showed reduced grain yield per plant.

**Conclusions:**

All the improved lines conferred wider resistance spectrum compared with the RP. Yet, the three monogenic lines did not work under field conditions of the two nurseries. Given the similar performances on the main agronomic traits as the RP, the two-gene pyramids have achieved the breeding goals of broad resistance spectrum and effective panicle blast resistance. Whereas, the three-gene pyramid harboring *Pi2*, *Pi46* and *Pita* seems superfluous considering its reduced yield, although it also showed displayed high level of blast resistance. Thus, rational use of *R* genes rather than stacking more *R* genes is recommended to control the disease.

## Background

Rice (*Oryza sativa* L.), a major staple food worldwide, feeds more than half of the world population. More than 90% of the world’s rice is produced and consumed in Asia (Khush 2005). Globally, China is the largest producer and consumer of rice (Wang et al. 2005). However, rice production encounters several constraints including biotic stresses, among which rice blast caused by the ascomycete *M. oryzae* is the most destructive. Commonly, the disease reduces yield by 10–30%, even up to 90% under favorable environmental conditions (Skamnioti and Gurr 2009). South China usually witnesses severe blast disease incidences, due to abundant rainfall and proper temperature during the rice growing season. It was reported that more than 1500 ha of rice was damaged by panicle blast disease in Leizhou City of Guangdong province, China in 2008, of which over 250 ha were completely yieldless (http://seed.aweb.com.cn/2008/1208/155631350.shtml). Moreover, nearly 10,000 ha of rice in Guangdong province, China was affected negatively by blast disease in 2016, with about 900 ha being damaged severely (http://kb.southcn.com/content/2016-05/06/content_147276723.htm). Though effective fungicide was used, fungicide application is not a sustainable, viable and bio-safe option for managing the disease.

Utilization of resistant cultivars has been universally considered as the most eco-friendly and sustainable approach to control rice blast (Ni et al. 2015). Previous studies have identified and mapped more than 100 different blast resistance (*R*) genes, and more than 30 have been cloned (Fukuoka et al. 2014, Ramkumar et al. 2015; Deng et al. 2017). Based on the molecular marker-assisted selection (MAS) approach, the identifications of *R* genes donors and linked molecular markers have greatly facilitated *R* genes transferring in rice breeding programs to improve resistance against blast disease (Khanna et al. 2015, Xiao et al. 2015). Moreover, some broad-spectrum resistant varieties were identified to harbor multiple *R* genes, including Tetep (Barman et al. 2004), IR64 (Sallaud et al. 2003), Sanhuangzhan 2 (Liu et al. 2004), Digu (Chen et al. 2004; Shang et al. 2009) and Gumei2 (Wu et al. 2005). These findings suggest that the combination of multiple race-specific *R* genes is an effective strategy to develop cultivars with broad-spectrum resistance to blast disease (Hittalmani et al. 2000, Tacconi et al. 2010, Khanna et al. 2015, Xiao et al. 2016). However, multiple *R* genes mean intensive pressure to promote the evolution of *M. oryzae* races, which is highly various in population due to a high level of genomic instability of the pathogen (Dean et al. 2005; Ballini et al. 2008). It is worried about that the super races could arise in an ecological region and result in severe blast epidemics via defeating the multiple major *R* genes. Therefore, it is crucial to slow down the evolution rate of the pathogen toward virulence for the plant disease managements (Ballini et al. 2008, Miah et al. 2013).

However, how to rationally utilize the race-specific *R* genes in a certain rice growing region to sustain the blast resistance of rice cultivars is still poorly understood. In this study, we developed three-gene pyramid harboring *Pi2*, *Pi46* and *Pita*, two-gene pyramids harboring either two of *Pi2*, *Pi46* and *Pita* and monogenic lines harboring either *Pi2*, *Pi46* or *Pita* by introgression of *R* gene(s) into a common genetic background using marker-assisted backcross breeding (MABB). Next, we assessed the disease resistance of these lines with one-to-three *R* gene(s) under artificial inoculation and field conditions at multi-locations. Lastly, we evaluated the agronomic traits of all the lines under conditions without blast disease stress. Our results showed that *Pi2*, *Pi46* and *Pita* three-gene pyramid may not be the best choice in the specific rice-growing regions of Guangdong province, China. The results will provide valuable insights for rice breeders to develop blast resistant rice varieties in a certain rice-growing region.

## Results

### MAS for foreground

**Fig. 1:**
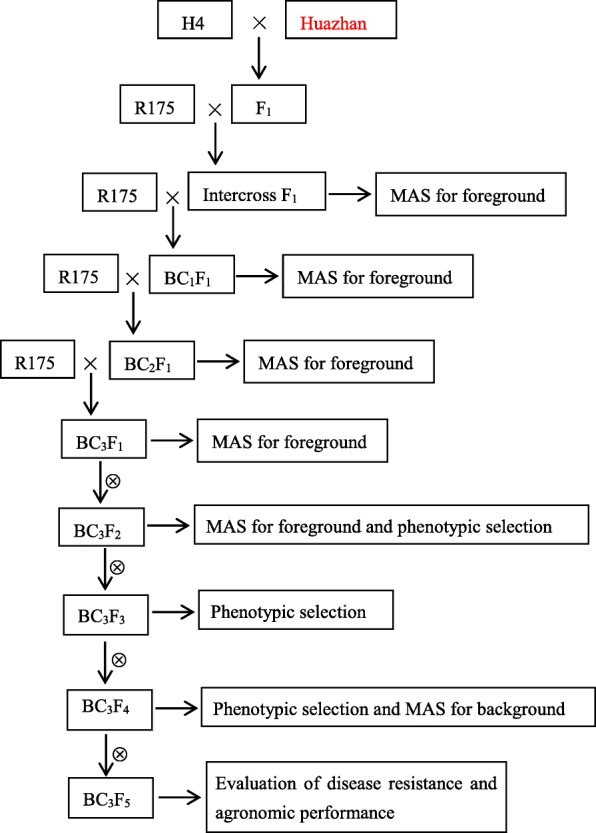
Schematic work flow of marker-assisted backcross breeding

In the early crop season of 2010, a total of 53 intercross F_1_ plants were identified for foreground selection using gene-based marker for *Pita* and gene-linked markers for *Pi46* and *Pi2* (Table 1). Six plants were found to be positive for the three target *R* genes with heterozygous state. Subsequently, the six positive plants were backcrossed with R175 using mixed pollen to generate the BC_1_F_1_ population. Finally, the advanced backcross progenies of BC_2_F_1_ and BC_3_F_1_ were obtained from the crosses of the selected resistant BC_1_F_1_ and BC_2_F_1_ plants based on the foreground selection (Fig. 1). Consequently, ten plants among the 82 BC_1_F_1_ individuals and seven plants among the 59 BC_2_F_1_ individuals were positive for the three loci with heterozygous state respectively. Among the 74 BC_3_F_1_ progenies, nine plants were identified to be heterozygous for *Pi46*, *Pi2* and *Pita* simultaneously. The BC_3_F_2_ population was produced from these nine individuals through self-pollination.

**Table 1:** Molecular markers used for foreground selection in this study

Marker	Type	Chr.	Forward primer sequence	Reverse primer sequence	Annealing temperature (°C)	Separation
RM224	SSR	11	ATCGATCGATCTTCACGAGG	TGCTATAAAAGGCATTCGGG	56	8% PAGE
Ind306	Indel	6	TGACTTCCAAACGGTAGC	AGAGCTCGTGAACGGAATG	56	8% PAGE
*Pita*-Ext	*Pita-*based marker	12	TGCGCAAAGAATCGTCGCTGC	TCTTTGATCCAAGTGTTAGGGCC	62	1% Agarose gel
*Pita*-Int		12	CCGTGGCTTCTATCTTTACCTG	AGTCAGGTTGAAGATGCATAGA	62

Initially, a total of 800 BC_3_F_2_ plants at the young seedling stage were genotyped using marker RM224 for the *Pi46* locus. As a result, 194 plants were homozygous for *Pi46*; 389 were heterozygous for *Pi46pi46* and 217 were homozygous for *pi46*, respectively. The segregation ratio of 194:389:217 was in accordance with 1:2:1 segregation according to the Chi-square test, *χ*^2^ = 1.93< *χ*^2^(0.05, 2) = 5.99. The 194 plants were then genotyped using Ind306 (for *Pi2*) and *Pita*-based marker, and the results of different genotypes were listed in Table 2. Therefore, we obtained ten monogenic plants (only *Pi46*), 27 di-genic plants (14 carrying *Pi46* + *Pi2* and 13 carrying *Pi46* + *Pita*) and eleven tri-genic plants (*Pi46* + *Pi2* + *Pita*) according to the results of foreground selection among the 194 plants. Meanwhile, the other 217 plants showing negative for *Pi46* were also genotyped using Ind306 and *Pita*-based marker to obtain the monogenic lines (*Pi2* or *Pita*) and two-gene pyramids (*Pi2* + *Pita*) (Table 3). As a result, fourteen plants carrying only *Pi2*, fifteen plants carrying only *Pita*, and twelve plants carrying both *Pi2* and *Pita* were obtained, respectively. Finally, seven types of plants with different *R* genes, including the three types of monogenic lines (*Pi46* or *Pi2* or *Pita*), three types of two-gene pyramids (*Pi46* + *Pi2*, *Pi46* + *Pita* and *Pi2* + *Pita*) and three-gene pyramids (*Pi46* + *Pi2* + *Pita*), were obtained from the BC_3_F_2_ progenies based on foreground selection.

**Table 2:** Results of genotypes based on Ind306 and *Pita*-based marker among the 194 *Pi46* homozygous plants

	*Pita* homozygous	*Pita* heterozygous	*pita* homozygous	Total	Segregation ratio for *Pita*	Segregation ratio for *Pi2*
*Pi2* homozygous	11^a^	22	14^b^	47	—	—
*Pi2* heterozygous	24	50	28	102	—	—
*pi2* homozygous	13^c^	22	10^d^	45	—	—
Total	48	94	52	194	48:94:52	47:102:45
*χ* ^2^	—	—	—	—	0.35	0.56

Note: ^a^ and ^d^ mean that the plants carried three *R* genes (*Pi46* + *Pi2* + *Pita*) and one *R* gene (*Pi46*), respectively; ^b^ and ^c^ mean that the plants carried two *R* genes, *Pi46* + *Pi2* and *Pi46* + *Pita*, respectively; *χ*^2^(0.05, 2) = 5.99.

**Table 3:** Results of genotypes based on Ind306 and *Pita*-based marker among the 217 *pi46* homozygous plants

	*Pita* homozygous	*Pita* heterozygous	*pita* homozygous	Total	Segregation ratio for *Pita*	Segregation ratio for *Pi2*
*Pi2* homozygous	12^a^	26	14^b^	52	—	—
*Pi2* heterozygous	28	56	21	105	—	—
*pi2* homozygous	15^c^	32	13	60	—	—
Total	55	114	48	217	55:114:48	52:105:60
*χ* ^2^	—	—	—	—	1.01	0.82

Note: ^a, b^ and ^c^ mean that the plants carried two *R* genes (*Pi2* + *Pita*), one *R* gene (*Pi2*) and one *R* gene (*Pita*), respectively; *χ*^2^(0.05, 2) = 5.99.

### Producing stable lines with different *R* gene(s) and MAS for background

To produce the seven types of stable lines with different *R* gene(s), we selected four plants displaying closest phenotypic resemblance to the RP from each type of the monogenic lines, two-gene pyramids and three-gene pyramids in the BC_3_F_2_ population at maturity stage. The main panicles of the four plants from each type of lines were collected and mixed as the corresponding BC_3_F_3_ offspring, respectively. Then, seven BC_3_F_3_ populations with each comprising 100 plants from the seven types of lines were planted adjacently with the RP. We also selected four plants showing the closest phenotypic resemblance to the RP from each population, collected their main panicles and mixed as the BC_3_F_4_ offspring. Likewise, we selected four plants from each of the seven BC_3_F_4_ populations and carried out background selection meanwhile, in that the agronomic traits became stable after consecutive self-crossing of several generations.

To carry out background selection efficiently, we firstly identified the polymorphic markers between the RP and DPs. Then the screened polymorphic markers were utilized to analyze the RP and the BC_3_F_1_ sample, which were mixed equally using the DNA from the nine BC_3_F_1_ plants positive for the three *R* genes with heterozygous state. The polymorphic markers reached 67 between R175 (RP) and H4 (DP), and 58 between R175 and Huazhan (DP), respectively. Considering the shared fifteen polymorphic markers, the total amount of polymorphic markers reached 110 (Additional file 1: Table S1). Among the 110 polymorphic markers, 96 markers showed monomorphic between the RP and the BC_3_F_1_ sample. Though the remaining fourteen markers showed polymorphic between the RP and the BC_3_F_1_ sample, all of them presented as heterozygous state. Therefore, the fourteen polymorphic markers (Additional file 1: Table S2) were utilized to detect the 28 plants selected from the seven BC_3_F_4_ populations. Among these remaining fourteen markers, the markers homozygous for the recipient allele were ranged from two to nine. Together with the 96 markers, the non-polymorphic markers reached a range of 98 to 105 among the 28 plants. It means that the genetic background recovery of the RP reached a range of 89.1 to 95.5%, with the average genome recovery of 92.89%. In addition, the average percentage of DPs genome was 4.38% with average residual heterozygosity of 2.69% (Additional file 1: Table S3). The plant in each population harboring the highest RP genome recovery and minimum residual heterozygosity was selected to produce advanced progenies by self-pollination, respectively. Finally, seven improved lines, named R501 (*Pi46*), R502 (*Pi2*), R503 (*Pita*), R504 (*Pi46* + *Pi2*), R505 (*Pi46* + *Pita*), R506 (*Pi2* + *Pita*) and R507 (*Pi46* + *Pi2* + *Pita*), were obtained for subsequent evaluation of blast resistance and assessment of agronomic traits (Additional file 1: Figure S1).

### Evaluation of blast resistance under artificial inoculation conditions

A total of 32 different *M. oryzae* isolates were used to evaluate the blast resistance of the seven improved lines, the RP and the DPs at the seedling stage. Common genetic background of the seven lines made it easier to test the resistance reactions of *Pi46*, *Pi2*, *Pita* and their combined effect. The results showed that both R504 and R507 conferred full-spectrum resistance, suggesting that they were resistant to all of the 32 isolates (Table 4). R505 and R506 showed the same resistance spectrum as 90.6%, followed by R502 (87.5%) and R501 (84.3%). Though R503 showed its resistance spectrum as 53.1%, which tremendously exceeded the RP (28.1%). Therefore, all of the seven improved lines, including the monogenic lines (R501, R502 and R503), the di-genic lines (R504, R505 and R506) and the tri-genic line (R507), conferred much wider resistance spectrum than the RP. Furthermore, an increasing trend was observed in the resistance spectrum from monogenic lines to di-genic lines and tri-genic line. Among the three monogenic lines, both R501 (*Pi46*) and R502 (*Pi2*) showed much broader resistance spectrum than R501 (*Pita*), implying R501 and R502 conferred resistance to more isolates than R503. Among the three di-genic lines, R504 (*Pi46* + *Pi2*) conditioned wider resistance spectrum than R505 (*Pi46* + *Pita*) and R506 (*Pi2* + *Pita*). The results elucidated that the combination of *Pi46* + *Pi2* could confer resistance to more *M. oryzae* isolates in this study. In addition, the resistance spectrum of the DPs (H4 and Huazhan) reached 100.0% and 87.5% respectively, indicating their excellent blast disease resistance in comparison to the RP.

**Table 4:** Resistance reactions of tested lines to 32 *M. oryzae* isolates

Isolates	Testing lines									
	R501 (*Pi46*)	R502 (*Pi2*)	R503 (*Pita*)	R504 (*Pi46*+*Pi2*)	R505 (*Pi46*+*Pita*)	R506 (*Pi2*+*Pita*)	R507 (*Pi46*+*Pi2*+*Pita*)	R175	H4 (*Pi46*+*Pita*)	Huazhan (*Pi2*)
GD93286	R	R	S	R	R	R	R	S	R	R
GD9866	R	R	S	R	R	R	R	S	R	S
GD00193	R	R	S	R	R	R	R	S	R	R
GD0526	R	R	R	R	R	R	R	R	R	R
GD0618	R	R	R	R	R	R	R	S	R	R
GD06141	R	R	S	R	R	R	R	S	R	R
GD06311	R	R	R	R	R	R	R	R	R	R
GD07116	R	S	R	R	R	R	R	S	R	S
GD07235	S	R	S	R	S	R	R	S	R	R
GD08866	R	R	R	R	R	R	R	R	R	R
GD08758	R	R	S	R	R	R	R	S	R	R
GD08950	R	R	S	R	R	R	R	S	R	R
GD08T6	S	R	R	R	R	R	R	S	R	R
GD08T13	S	R	S	R	S	R	R	S	R	R
GD0983	R	R	R	R	R	R	R	R	R	R
GD09103	R	R	S	R	R	R	R	S	R	R
GD09109	R	R	R	R	R	R	R	R	R	R
GD09305	R	R	S	R	R	R	R	S	R	R
GD10112	R	R	R	R	R	R	R	R	R	R
GD10127	R	S	S	R	R	S	R	S	R	S
GD10318	R	R	R	R	R	R	R	S	R	S
GD10359	S	R	R	R	R	R	R	S	R	R
GD10405	R	R	R	R	R	R	R	R	R	R
GD10424	R	R	R	R	R	R	R	S	R	R
GD10431	R	S	S	R	R	S	R	S	R	S
GD10555	R	R	S	R	R	R	R	S	R	R
GD10560	R	R	R	R	R	R	R	S	R	R
GD11122	R	R	R	R	R	R	R	R	R	R
GD11161	S	R	S	R	S	R	R	S	R	R
GD11235	R	R	R	R	R	R	R	R	R	R
GD11318	R	R	R	R	R	R	R	S	R	R
GD12306	R	S	S	R	R	S	R	S	R	S
Resistance spectrum	84.3%	87.5%	53.1%	100.0%	90.6%	90.6%	100.0%	28.1%	100.0%	87.5%

Note: Resistance spectrum was calculated from the ratio of the number of incompatible isolates and the total number of tested isolates. *R* Resistant, *S* Susceptible.

### Evaluation of panicle blast resistance at natural nurseries

To evaluate the blast resistance comprehensively, the panicle blast resistance of the seven improved lines was assessed under field conditions of two natural nurseries respectively (Table 5). All the three monogenic lines showed severer infected panicles with higher ratios compared with the di-genic lines and the tri-genic line. Among the three monogenic lines, R503 (*Pita*) displayed the same symptom as the RP and fully succumbed to panicle blast disease at two locations. The other two monogenic lines R501 (*Pi46*) and R501 (*Pi2*) showed severe panicle blast, although their ratings and ratios of infected panicles ranked below those of R503 and the RP. Hence, the monogenic lines cannot work under field conditions of the two nurseries. Among the three two-gene pyramids, R504 showed slight rating and low ratio of infected panicle, followed by R505 and R506. Whereas, R505 and R506 presented the same rating and ratio of infected panicles at two locations, indicating that they showed the same level of resistance to panicle blast. The three-gene pyramid R507 conferred good panicle blast resistance, displaying the same rating and ratio of infected panicles as those of the two-gene pyramid R504 and the DP H4. The other DP Huazhan conferred moderate panicle blast resistance, ranking between the di-genic lines and the monogenic lines as for the rating and ratio of infected panicles. On the other hand, it was found that the ratios of infected panicles at Conghua nursery were higher than those at Yangjiang nursery (Table 5), suggesting that the conditions at Conghua nursery facilitated the development of the disease. In general, the panicle blast resistance was distinctly different among the seven improved lines, the RP and the DPs at natural nurseries.

**Table 5:** Panicle blast resistance of tested lines under field conditions

Lines	Conghua		Yangjiang	
	Rating of severely infected panicles	Ratio of severely infected panicles	Rating of severely infected panicles	Ratio of severely infected panicles
R501 (*Pi46*)	7^a^	90%	5^a^	70%
R502 (*Pi2*)	7	70%	5	50%
R503 (*Pita*)	9	100%	9	100%
R504 (*Pi46*+*Pi2*)	1	5%	1	3%
R505 (*Pi46*+*Pita*)	3	10%	3	5%
R506 (*Pi2*+*Pita*)	3	10%	3	5%
R507 (*Pi46*+*Pi2*+*Pita*)	1	5%	1	3%
R175	9	100%	9	100%
H4 (*Pi46*+*Pita*)	1	5%	1	3%
Huazhan (*Pi2*)	5	20%	5	15%

Note: ^a^ the ratings of panicle blast disease. 0: no incidence of infected grains; 1: infected symptoms only at spikelets; 3: infected symptoms expand to second branches of a panicle; 5: infected symptoms expand to first branches of a panicle; 7: infected symptoms expand to the main branch of a panicle; 9: the whole panicle is infected severely.

### Assessment of agronomic traits

**Fig. 2:**
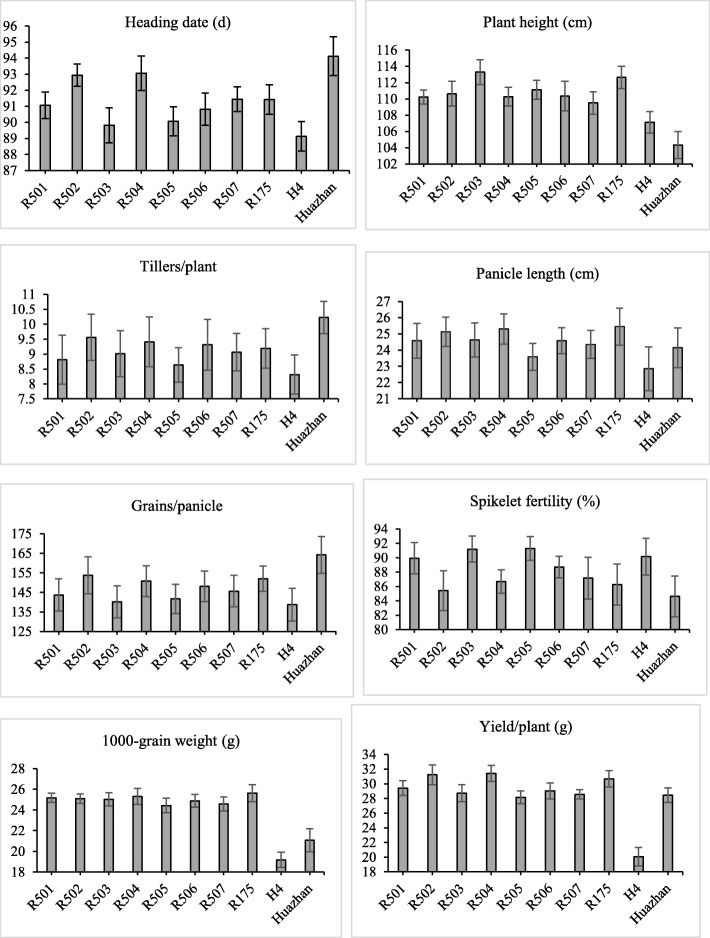
Performances of main agronomic traits of the tested lines during the early crop season (March to July) of 2016

To compare the performances on the agronomic traits among the tested lines, a set of monogenic, di-genic and tri-genic lines together with the RP and DPs were planted in Guangzhou where there was no disease stress. For most of the traits, the improved lines showed phenotypic resemblance to the RP. However, the improved lines showed significant difference from the RP in several traits (Additional file 1: Table S4). In comparison to the RP, the improved version R501 reduced its plant height significantly, but increased its spikelet fertility significantly. Both R502 and R504 showed significantly increased heading date but also reduced plant height significantly. R503 had significantly decreased heading date, grains per panicle and grain yield, but highly significant raise in spikelet fertility. Regarding R505, highly significant reduction in grain yield, but significant decrease in heading date, panicle length, grains per panicle and 1000-grain weight, and highly significant increase in spikelet fertility, were observed. As to R506, significant reductions in plant height and grain yield were observed. Apart from significant decrease in 1000-grain weight, R507 also showed highly significant reduction in plant height and grain yield. In summary, the improved lines were different from the RP in at least two traits among the seven improved lines. For example, some improved lines could mature early, some became dwarf, and others had high spikelet fertility. As to the donor parent H4, it had highly significant reduction in heading date, plant height, panicle length, 1000-grain weight and grain yield and significant decrease in grains per panicle in comparison to the RP. Regarding the donor parent Huazhan, it showed highly significant increase in heading date and significant raise in tillers per plant and grains per panicle, but highly significant reduction in plant height, 1000-grain weight and grain yield compared with the RP. To display the traits visually, the data of each trait for the improved lines, RP and DPs are showed as Fig. 2.

## Discussion and conclusions

Rice blast, as a major biological stress, has a serious impact on rice production. Thus, the improvement of rice blast resistance has become one of the most important breeding targets. However, phenotype identification of rice blast resistance is difficult and unstable in different years and locations. Additionally, it is difficult to pyramid multiple *R* genes through conventional breeding strategy in the case when the resistance reactions of one *R* gene could be masked by other *R* genes (Koide et al. 2011). The both disadvantages can be overcome through MAS for its efficiency and effectiveness (Suh et al. 2011; Mi et al. 2018). Based on the MAS approach, several advanced breeding lines/varieties for blast resistance have been developed successfully (Khanna et al. 2015; Ellur et al. 2016; Xiao et al. 2018).

Actually, the accuracy of MAS is mainly determined by the distance between the target gene and the marker. In this study, we used the marker *Pita*-Ext/Int for MAS of *Pita*. Since *Pita*-Ext/Int is a functional marker of the *Pita* gene (Ramkumar et al. 2015). Thus, there is no doubt about the accuracy of MAS. As for *Pi46*, we used the SSR marker RM224 for the MAS of the *R* gene. Because the marker is linked with *Pi46* at ~ 1.0 cM (Xiao et al. 2011), which is close enough for MAS. In addition, the chromosome region where *Pi46* resides in is a cross-cold region, and this feature suppresses recombination between the *R* gene and the marker RM224 (Xu et al. 2008; Xiao et al. 2011). The marker Ind306 locates at ~ 29.0 kb upstream of the gene *Pi2*, which is actually very close to *Pi2* for MAS. Our previous research has verified the satisfying effectiveness of MAS for *Pi2* using the marker Ind306 (data not shown). All the improved versions, no matter the monogenic lines or the two-gene pyramids or the three-gene pyramid, showed broader spectrum of blast resistance at seedling stage and enhanced resistance level to panicle blast as compared to the RP. These results confirmed the accuracy of MAS for the three genes *Pi46*, *Pi2* and *Pita* using the corresponding markers RM224, Ind306 and *Pita*-Ext/Int, respectively.

Incorporation of major *R* genes has been recognized as one of the most effective strategies for managing rice blast disease. Nonetheless, a resistant variety carrying only single major gene may be rapidly overcome by virulent races of the pathogen which is highly variable during the course of commercial utilization (Xiao et al. 2017). Therefore, it is proposed to pyramid the genes for attaining durable resistance (Sreewongchai et al. 2010; Rabion et al. 2016). However, is it really reasonable and necessary to develop resistant cultivars by pyramiding as much as possible *R* genes in a specific ecological region? In other words, the pyramids with more *R* genes are really better than those with less *R* genes?

To deepen our understanding to this question, particularly in the breeding practice for rice blast disease in Guangdong province, China, we developed seven improved lines (named from R501 to R507 respectively) carrying one to three *R* genes purposely in this study. In fact, the seven lines could be recognized as a set of near-isogenic lines (NILs), considering their common genetic background. Therefore, the NILs benefit to the comparison of resistance reactions of the *R* genes and their combinations, because the disturbance from other genetic backgrounds could be eliminated among the NILs. The results showed that the two-gene pyramids and the three-gene pyramid were absolutely better than the monogenic lines whether on the resistance spectrum at seeding stage under artificial conditions or on the panicle blast resistance at adult stage under filed conditions. However, the two-gene pyramid R504 and the three-gene pyramid R507 not only showed full spectrum of resistance under artificial inoculation conditions, they also showed the same level of resistance to panicle blast at the two natural nurseries. It implied that the two-gene pyramid R504 can reach the resistance effect of the three-gene pyramid R507. Indeed, it is inevitable to bring more genomic compositions from the DPs into the RP to produce three-gene pyramid than two-gene pyramid in the same procedure (Luo et al. 2017). Eventually, it is confirmed that R507 exhibited low RP genome recovery and differences on more traits from RP as compared to R504.

Generally, the risk of co-introduction of undesirable characteristics increases according to the number of *R* gens in backcross breeding. In this study, large chromosomal segment was introgressed into the genome of RP along with the introduction of *Pita* from the DP, because *Pita* resides in the pericentromeric region which is of low recombination rates (Jia and Martin, 2008; Xia et al. 2012). Some loci controlling panicle traits, including panicle length, spikelets per panicle and seed setting density, are reported to locate in the region near the centromere of chromosome 12 (Wu et al. 2016). The reduced grain yield of R503, R505, R506 and R507 may be caused by the loci linked with *Pita* when the large segment was introgressed and other yield-related loci from DPs. All the improved lines R501, R502, R504, R506 and R507 showed reduced plant height, they carried a common segment around marker RM497 on chromosome 2. The reduced plant height of these lines may be resulted from the segment, which may harbor the gene(s) affecting plant height. In addition, other gene(s) or QTL allele(s) derived from DPs controlling plant height could not be excluded, considering that the plant height of the two DPs were extremely reduced compared with the RP. Though R502 and R504 showed significant delayed heading date, they did not show significant high grains and grain yield in comparison to the RP. The phenomenon could be partially explained by the unidentified late heading QTL allele(s) other than *Ghd7* and *Ghd8* which regulates plant height, heading date and grain yield in rice (Xue et al. 2008; Yan et al. 2011). Since the marker density for the background selection in this work is relatively low, the genetic background of the improved lines could not be revealed in full detail. The rough results of background analysis could not fully show the chromosomal segments which were introgressed into the RP genome from the DPs. Nonetheless, the segments, which carry gene(s) or QTL allele(s) controlling agronomic traits, were introduced into the RP genome from the DPs in the course of MABB. These introduced segments may account for the different traits of the improved lines. Taking account of low genome recovery and agronomic traits, the three-gene pyramid is not better than the two-gene pyramids in this study.

The three-gene pyramid R507 was still infected under field conditions, though it showed low ratio of infected panicles and conferred resistance to all the races under artificial conditions. It indicated that the races are more complicated under field conditions and some races can conquer the three *R* genes. It also revealed that R507 is likely to lose its resistance once the virulent races become dominant. Under such circumstances, the two-gene pyramids (carrying *Pi46* + *Pi2*, *Pi46* + *Pita* and *Pi2* + *Pita*, respectively) will no longer work effectively. To avoid the emergence and spread of more virulent races, rational use of *R* genes should be concerned to slow down the evolution of the races (Kwon et al. 2008; Telebanco-Yanoria et al. 2011). In this study, the two-gene pyramids R504, R505 and R506 just showed slight susceptibility at field hotspots. It means that they could be utilized in wide rice production area because they harbored the combination of several key *R* genes, which still remain effective in the southern China rice production area (Zhang et al. 2017). Furthermore, large parts of ecological regions in southern China are not like the hot spot natural nurseries, which is specifically favorable for the development of blast disease. Taken together, it is recommended that rational use of *R* genes rather than stacking more *R* genes to control the disease.

## Materials and methods

### Plant materials and MABB procedure

In this study, two *indica* accessions, H4 and Huazhan, were used as donor parents (DPs). The accession H4, conferring broad-spectrum resistance to blast at both seedling and adult stages, was found to carry at least two major *R* genes, with *Pi46* on the long arm of chromosome 11 (Xiao et al. 2011) and *Pita* on chromosome 12 (Xiao et al. 2016). The *R* gene *Pi46* was confirmed to be a different allele of *Pik*/*Pi1* locus, for several SNPs that could discriminate them were identified (data not shown). Huazhan, a famous restoring line which has being utilized widely in China in recent years, was found to harbor *Pi2* on chromosome 6 (Tian et al. 2016). R175, an elite *indica* restoring line with good grain quality, was used as the RP because of its poor blast resistance.

Firstly, we produced an F_1_ population of Huazhan/H4 to pyramid the three above-mentioned *R* genes. An intercross F_1_ population was subsequently generated by crossing the F_1_ plants (Huazhan/H4) with R175. Then the backcrossing procedure was followed using R175 as the RP till to BC_3_F_1_ generation. The plants showing positive for the three target *R* genes and harboring the maximum recurrent parent genome (RPG) recovery were identified. These identified plants were used to produce advanced generations through pedigree method of selection. The different generations were handled as per the MABB scheme presented in Fig. 1. The scheme consisted of a two-step strategy in each backcross generation: (1) foreground selection for the target *R* gene(s) using gene-based/linked markers; (2) stringent phenotypic selection for agronomic traits to accelerate the RPP recovery.

### Molecular marker analysis

Regarding the foreground selection, gene-linked markers RM224 and Ind306 were used for the genes *Pi46* and *Pi2*, respectively. The selection for the gene *Pita* was conducted using gene-based markers *Pita-*Ext/Int according to the method reported by Ramkumar et al. (2015). Marker RM224 is tightly linked with *Pi46* at ~ 1.0 cM (Xiao et al. 2011). Whereas, marker Ind306 locates at ~ 29.0 kb upstream of the gene *Pi2*. This marker was developed several years ago and has being utilized in the research group. The information of the markers mentioned above is listed in Table 1.

For further background selection, the other 302 SSR markers, distributing evenly on the twelve rice chromosomes, were used for polymorphism survey between the DPs and RP. Subsequently, the polymorphic SSR markers were used to recover the RPG. The background selection was firstly performed using polymorphic markers in the BC_3_F_1_ population and then the remaining polymorphic markers were employed for the BC_3_F_4_ population. Genomic DNA was extracted from frozen leaf materials using the CTAB method (Murray and Thompson 1980) with minor modifications. Each 20 μL PCR reaction consisted of 1× PCR buffer (10 mmol L^− 1^ Tris, pH 8.4, 50 mmol L^− 1^ KCl, 1.8 mmol L^− 1^ MgCl_2_), 0.05 mmol L^− 1^ dNTPs, 5 pmol of each primer, 1.0 U of *Taq* polymerase, and 50 ng genomic DNA. All amplifications were performed using an ABI thermal cycler under the following profile: 94 °C for 5 min; 32 cycles of 30 s at 94 °C, 30 s at 55 °C, and 1 min at 72 °C; and an extension of 5 min at 72 °C. The PCR products of markers RM224 and Ind306 were separated in 8% non-denatured polyacrylamide gel (PAGE) in 1.0× TBE buffer followed by silver stains. Whereas, the PCR products of marker *Pita-*Ext/Int were visualized by gel electrophoresis on a 1% (w/v) agarose gel in 1.0× TBE buffer (Ramkumar et al. 2015).

### Evaluation for disease resistance under artificial inoculation

The three-gene pyramid, two-gene pyramids, monogenic lines and RP were tested for resistance spectrum by artificial inoculation at the seedling stage. In total, 32 highly diverse *M. oryzae* isolates, which were collected from different ecological areas in Guangdong Province, China during more than 10 years, were used for artificial inoculation to test leaf blast resistance. Two-week-old seedlings were spray-inoculated with spore suspensions (1 × 10^5^ spores mL^− 1^) and were cultured in a dew growth chamber for 24 h in darkness at 26 °C. The inoculated seedlings were subsequently transferred into a semi-temperature-controlled greenhouse where the temperature and relative humidity were maintained for 6 days at around 24–30 °C and 90%, respectively. The most serious disease lesions on the inoculated rice leaves were rated on a scale of 0–9, with rating of 0–3 considered as resistant (R) and 4–9 as susceptible (S) according to the Standard Evaluation System for Rice (International Rice Research Institute 2013).

### Multi-location evaluation of disease resistance under field conditions

To evaluate panicle blast resistance, the tested lines were grown at two hot spot natural nurseries, Conghua (23.57°N, 113.55°E) in the middle of Guangdong Province, and Yangjiang (21.50°N, 111.58°E) in the south of the province, during the early crop season (March to July) of 2016. Both sites are characterized with the microclimate (proper temperature and high humidity) favorable for outbreaks of numerous *M. oryzae* races during the rice growing season. Each entry was planted in four rows with five plants per row at a planting density of 20 cm × 20 cm. To maintain the pathogen population diversity and to enhance natural infection, each plot was surrounded by the highly susceptible variety CO39 as a spreader. Panicle blast resistance was measured using the 0–9 scale of the Standard Evaluation System for Rice (International Rice Research Institute 2013).

### Evaluation of agronomic traits

The monogenic, di-genic and tri-genic lines along with the RP R175 were planted using a randomized complete block design with three replications at the experimental field of South China Agricultural University, Guangzhou, during the early crop season (March to July) of 2016. Each plots consisted of six rows with six plants per row at a planting density of 20 cm × 20 cm. Only four plants in the middle of each plot were used to measure agronomic traits, including the heading date (HD) (days to 50% flowering), plant height (PH), tillers per plant (TP), panicle length (PL), total grains per panicle (TGP), spikelet fertility (SF), 1000-grain weight (TGW), and grain yield per plant (GY). Water and fertilizer were managed regularly. Statistical analysis was performed with independent samples using the least significance difference (LSD) software.

## Supplementary information

**Additional file 1: Table S1.** The 110 polymorphic markers between the RP and the DPs. **Table S2.** The information of the remaining 14 markers showing the DPs’ genotype at homozygous or heterozygous state. **Table S3.** The percentage of parental genome recovery of the 28 BC_3_F_4_ plants. **Table S4.** Performances of main agronomic traits of the tested lines during the early crop season (March to July) of 2016. **Figure S1.** Genetic background analysis of the seven improved lines by the remaining 14 SSR markers which detected polymorphic between the RP and the BC_3_F_1_ sample.

## Data Availability

The datasets supporting the conclusions of this article are provided within the article and its additional files.

## Abbreviations

DP: Donor parent
*M. oryzae*: *Magnaporthe oryzae*
MABB: Marker-assisted backcross breeding
MAS: Molecular assisted selection
NILs: Near-isogenic lines
PAGE: Polyacrylamide gel electrophoresis
R: Resistance
RGP: Recurrent parent genome
RP: Recurrent parent
