# Supplementary material for: Improvement of rice blast resistance by developing monogenic lines, two-gene pyramids and three-gene pyramid through MAS
Source: Rice (N Y). 2019 Nov 4;12:78. doi: 10.1186/s12284-019-0336-4 (PMC6828908; doi:10.1186/s12284-019-0336-4)
Supplement: Supplementary file 1 — Additional file 1: Table S1. The 110 polymorphic markers between the RP and the DPs. Table S2. The information of the remaining 14 markers showing the DPs’ genotype at homozygous or heterozygous state. Table S3. The percentage of parental genome recovery of the 28 BC3F4 plants. Table S4. Performances of main agronomic traits of the tested lines during the early crop season (March to July) of 2016. Figure S1. Genetic background analysis of the seven improved lines by the remaining 14 SSR markers which detected polymorphic between the RP and the BC3F1 sample. [file 12284_2019_336_MOESM1_ESM.docx]

Table S1 The 110 polymorphic markers between the RP and the DPs

| Chromosome | Markers |
| --- | --- |
| 1 | RM462, RM428, RM272, RM243, RM493, RM306, RM246, RM128, RM486, RM472, RM529 |
| 2 | RM154, RM279, RM555, RM492, RM475, RM526, RM497, RM208, RM535 |
| 3 | RM132, RM545, RM218, RM563, RM282, RM156, RM503, RM135, RM468, RM442 |
| 4 | RM401, RM335, RM261, RM417, RM273, RM451, RM303, RM348, RM124 |
| 5 | RM159, RM413, RM574, RM516, RM430, RM440, RM421, RM480, |
| 6 | RM508, RM587, RM204, RM314, RM549, RM527, RM564 (RM19877), RM162, RM30, RM103 |
| 7 | RM436, RM481, RM125, RM214, RM418, RM336, RM234, RM248 |
| 8 | RM407, RM38, RM25, RM547, RM404, RM223, RM256, RM230, RM477 |
| 9 | RM285, RM219, RM321, RM409, RM434, RM257, RM328, RM205 |
| 10 | RM474, RM216, RM239, RM467, RM184, RM258, RM304, RM228, RM591 |
| 11 | RM286, RM167, RM120, RM479, RM202, RM287, RM457, RM254, RM206, RM144 |
| 12 | RM415, RM453, RM491, RM512, RM179, RM519, RM463, RM270, RM17 |

Table S2 The information of the remaining 14 markers showing the DPs’ genotype at homozygous or heterozygous state

| Marker No. | Marker name | Chromosome | Position (Mb) |
| --- | --- | --- | --- |
| 1 | RM490 | 1 | 6.677 |
| 2 | RM497 | 2 | 29.034 |
| 3 | RM336 | 7 | 21.872 |
| 4 | RM467 | 10 | 13.560 |
| 5 | RM479 | 11 | 7.697 |
| 6 | RM144 | 11 | 28.804 |
| 7 | RM218 | 3 | 8.406 |
| 8 | RM527 | 6 | 9.863 |
| 9 | RM564 (RM19877) | 6 | 11.768 |
| 10 | RM223 | 8 | 20.652 |
| 11 | RM472 | 1 | 37.890 |
| 12 | RM451 | 4 | 27.042 |
| 13 | RM430 | 5 | 18.781 |
| 14 | RM179 | 12 | 14.401 |

Table S3 The percentage of parental genome recovery of the 28 BC_3_F_4_ plants

| Plants | RP genome (%) | DPs genome (%) | Residual heterozygosity (%) | Plants | RP genome (%) | DPs genome (%) | Residual heterozygosity (%) |
| --- | --- | --- | --- | --- | --- | --- | --- |
| 1 (*Pi46* only) | 93.6 (103^a^) | 3.64 (4^b^) | 2.73 (3^c^) | 15 (*Pi46*+*Pi2*) | 92.7 (102 ^a^) | 4.55 (5 ^b^) | 2.73 (3 ^c^) |
| 2 (*Pi46* only) | 93.6 (103) | 4.55 (5) | 1.82 (2) | 16 (*Pi46*+*Pi2*) | 93.6 (103) | 3.64 (4) | 2.73 (3) |
| 3 (*Pi46* only) | 92.7 (102) | 4.55 (5) | 2.73 (3) | 17 (*Pi46*+*Pita*) | 92.7 (102) | 4.55 (5) | 2.73 (3) |
| 4 (*Pi46* only) | 94.5 (104) | 3.64 (4) | 1.82 (2) | 18 (*Pi46*+*Pita*) | 91.8 (101) | 4.55 (5) | 3.64 (4) |
| 5 (*Pi2* only) | 93.6 (103) | 4.55 (5) | 1.82 (2) | 19 (*Pi46*+*Pita*) | 91.8 (101) | 5.45 (6) | 2.73 (3) |
| 6 (*Pi2* only) | 95.5 (105) | 2.73 (3) | 1.82 (2) | 20 (*Pi46*+*Pita*) | 92.7 (102) | 4.55 (5) | 2.73 (3) |
| 7 (*Pi2* only) | 93.6 (103) | 3.64 (4) | 2.73 (3) | 21 (*Pi2*+*Pita*) | 92.7 (102) | 4.55 (5) | 2.73 (3) |
| 8 (*Pi2* only) | 94.5 (104) | 3.64 (4) | 1.82 (2) | 22 (*Pi2*+*Pita*) | 92.7 (102) | 5.45 (6) | 1.82 (2) |
| 9 (*Pita* only) | 93.6 (103) | 2.73 (3) | 3.64 (4) | 23 (*Pi2*+*Pita*) | 93.6 (103) | 3.64 (4) | 2.73 (3) |
| 10 (*Pita* only) | 94.5 (104) | 2.73 (3) | 2.73 (3) | 24 (*Pi2*+*Pita*) | 91.8 (101) | 4.55 (5) | 3.64 (4) |
| 11 (*Pita* only) | 92.7 (102) | 4.55 (5) | 2.73 (3) | 25 (*Pi46*+ *Pi2*+*Pita*) | 89.1 (98) | 7.27 (8) | 3.64 (4) |
| 12 (*Pita* only) | 93.6 (103) | 3.64 (4) | 2.73 (3) | 26 (*Pi46*+ *Pi2*+*Pita*) | 91.8 (101) | 5.45 (6) | 2.73 (3) |
| 13 (*Pi46*+*Pi2*) | 91.8 (101) | 5.45 (6) | 2.73 (3) | 27 (*Pi46*+ *Pi2*+*Pita*) | 90.9 (100) | 5.45 (5) | 3.64 (4) |
| 14 (*Pi46*+*Pi2*) | 93.6 (103) | 4.55 (5) | 1.82 (2) | 28 (*Pi46*+ *Pi2*+*Pita*) | 91.8 (101) | 4.55 (5) | 3.64 (4) |
|  |  |  |  | Mean | 92.9 (102.21) | 4.38 (4.82) | 2.69 (2.96) |

Note: ^a, b^ and ^c^ mean the markers that showed the RP genotype, DPs genotype and heterozygosity, respectively. The plants that were underlined were selected to produce advanced progenies by self-pollination for further study.

Table S4 Performances of main agronomic traits of the tested lines during the early crop season (March to July) of 2016

| Lines | Heading date | Plant height (cm) | Tillers per plant | Panicle length (cm) | Grains per panicle | Spikelet fertility (%) | 1000-grain weight (g) | Grain yield per plant (g) |
| --- | --- | --- | --- | --- | --- | --- | --- | --- |
| R501 (*Pi46*) | 91.06±0.83 | 110.23±0.95^*^ | 8.81±0.82 | 24.57±1.06 | 143.67±8.26 | 89.93±2.17^*^ | 25.18±0.45 | 29.41±1.01 |
| R502 (*Pi2*) | 92.94±0.69^*^ | 110.64±1.52^*^ | 9.56±0.77 | 25.13±0.90 | 153.75±9.49 | 85.43±2.76 | 25.08±0.46 | 31.23±1.33 |
| R503 (*Pita*) | 89.81±1.08^*^ | 113.30±1.53 | 9.01±0.77 | 24.63±1.05 | 140.18±8.18^*^ | 91.20±1.81^**^ | 25.03±0.63 | 28.71±1.15^*^ |
| R504 (*Pi46*+*Pi2*) | 93.06±1.07^*^ | 110.28±1.17^*^ | 9.41±0.84 | 25.30±0.93 | 150.76±7.83 | 86.68±1.63 | 25.30±0.77 | 31.40±1.11 |
| R505 (*Pi46*+*Pita*) | 90.06±0.90^*^ | 111.12±1.18 | 8.64±0.58 | 23.58±0.83^*^ | 141.69±7.48^*^ | 91.30±1.66^**^ | 24.43±0.70^*^ | 28.15±0.87^**^ |
| R506 (*Pi2*+*Pita*) | 90.81±1.01 | 110.35±1.81^*^ | 9.31±0.85 | 24.58±0.81 | 148.08±7.77 | 88.73±1.50 | 24.88±0.62 | 29.03±1.08^*^ |
| R507 (*Pi46*+*Pi2*+*Pita*) | 91.44±0.77 | 109.50±1.38^**^ | 9.06±0.63 | 24.35±0.87 | 145.64±8.10 | 87.15±2.90 | 24.58±0.68^*^ | 28.55±0.63^**^ |
| R175 | 91.43±0.92 | 112.65±1.38 | 9.19±0.66 | 25.45±1.14 | 152.01±6.46 | 86.27±2.85 | 25.63±0.83 | 30.68±1.12 |
| H4 (*Pi46*+*Pita*) | 89.13±0.92^**^ | 107.13±1.31^**^ | 8.31±0.66 | 22.85±1.35^**^ | 138.69±8.33^*^ | 90.15±2.57 | 19.18±0.73^**^ | 20.08±1.26^**^ |
| Huazhan (*Pi2*) | 94.13±1.20^**^ | 104.33±1.65^**^ | 10.23±0.54^*^ | 24.15±1.23 | 164.18±9.41^*^ | 84.62±2.84 | 21.08±1.12^**^ | 28.45±0.98^**^ |

Note: Data are means ± SD. *and ** means that the agronomic trait showed significant difference when compared with the RP at P<0.05 and P<0.01 levels, respectively.


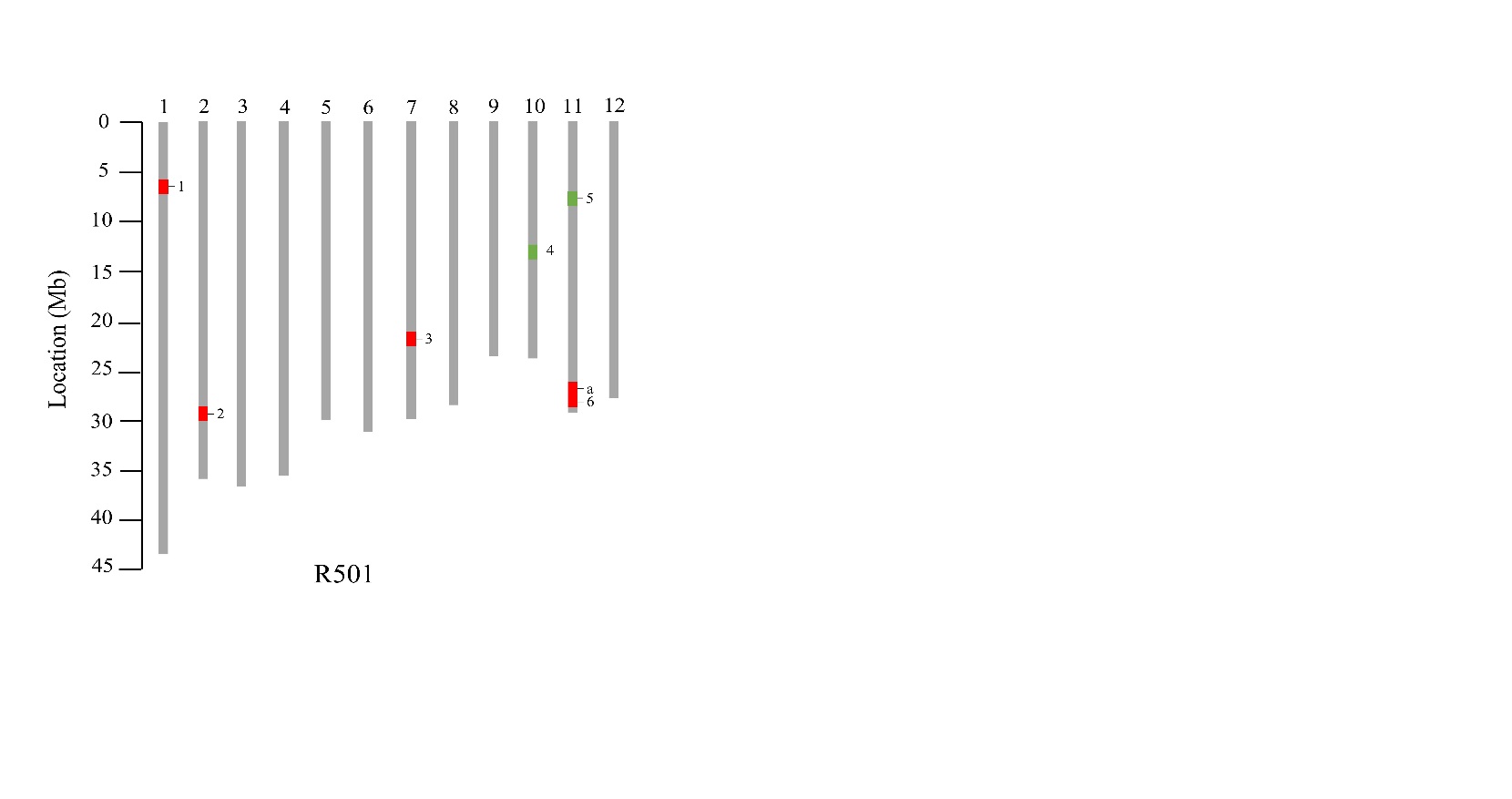

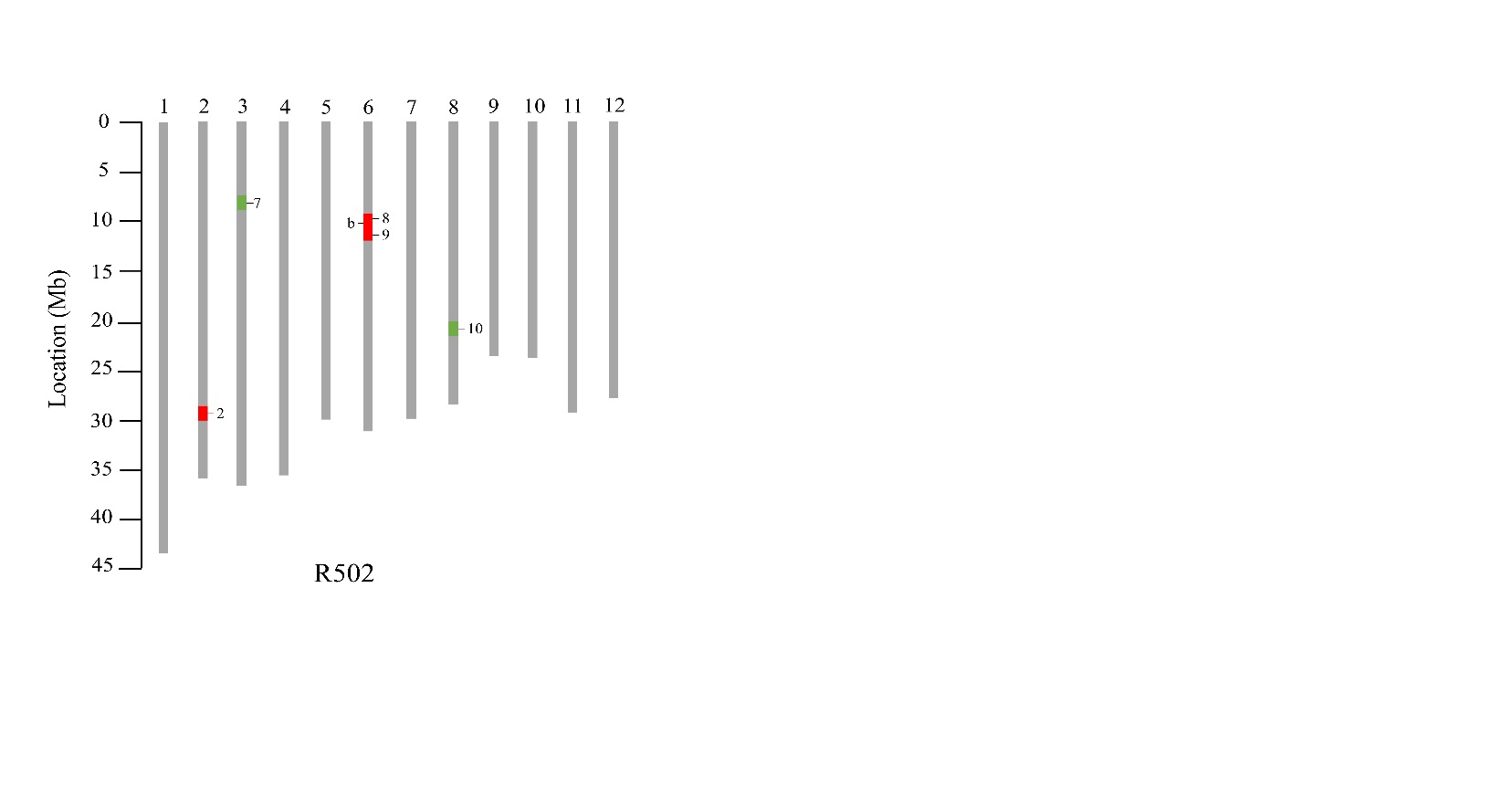


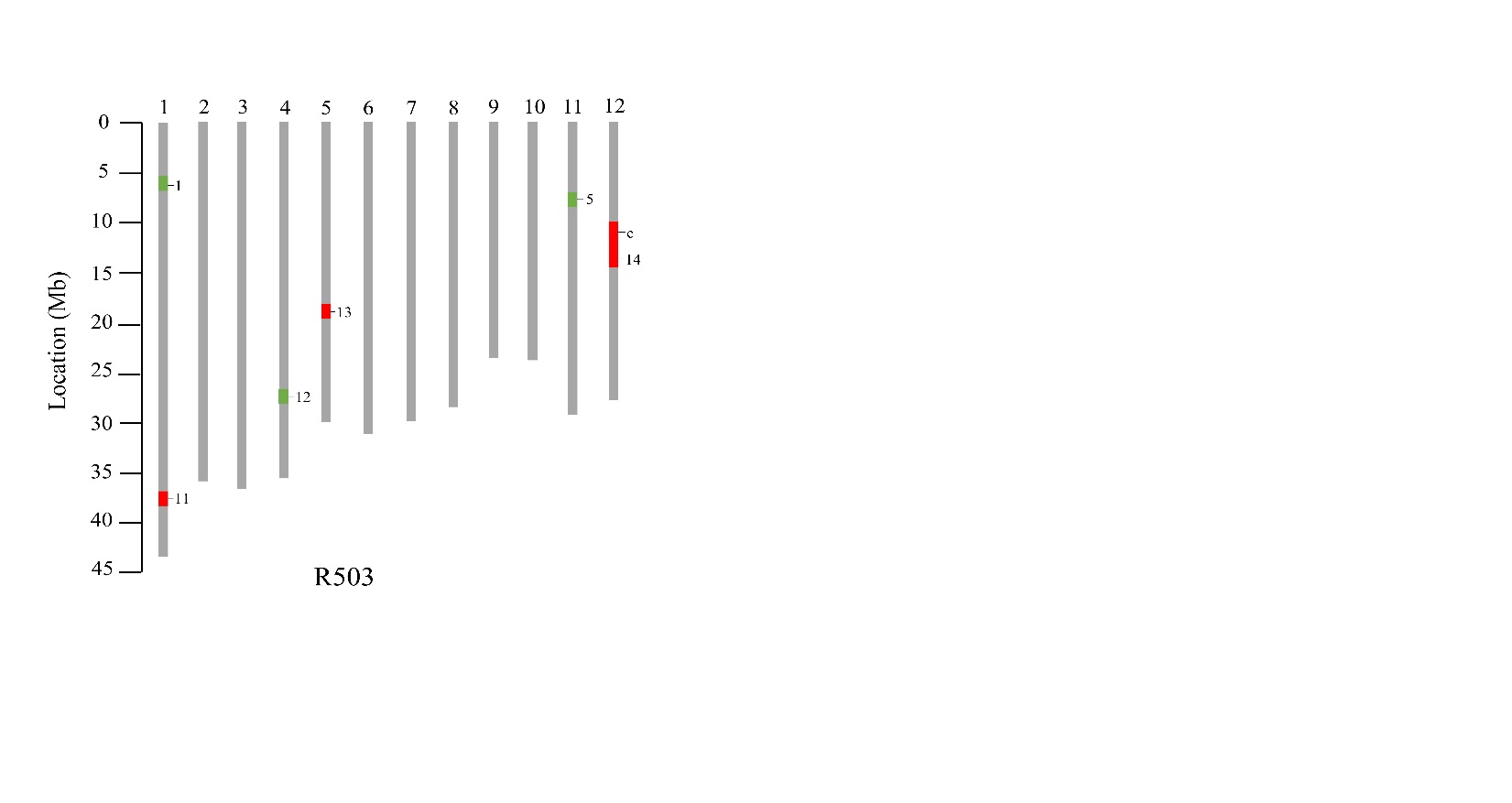

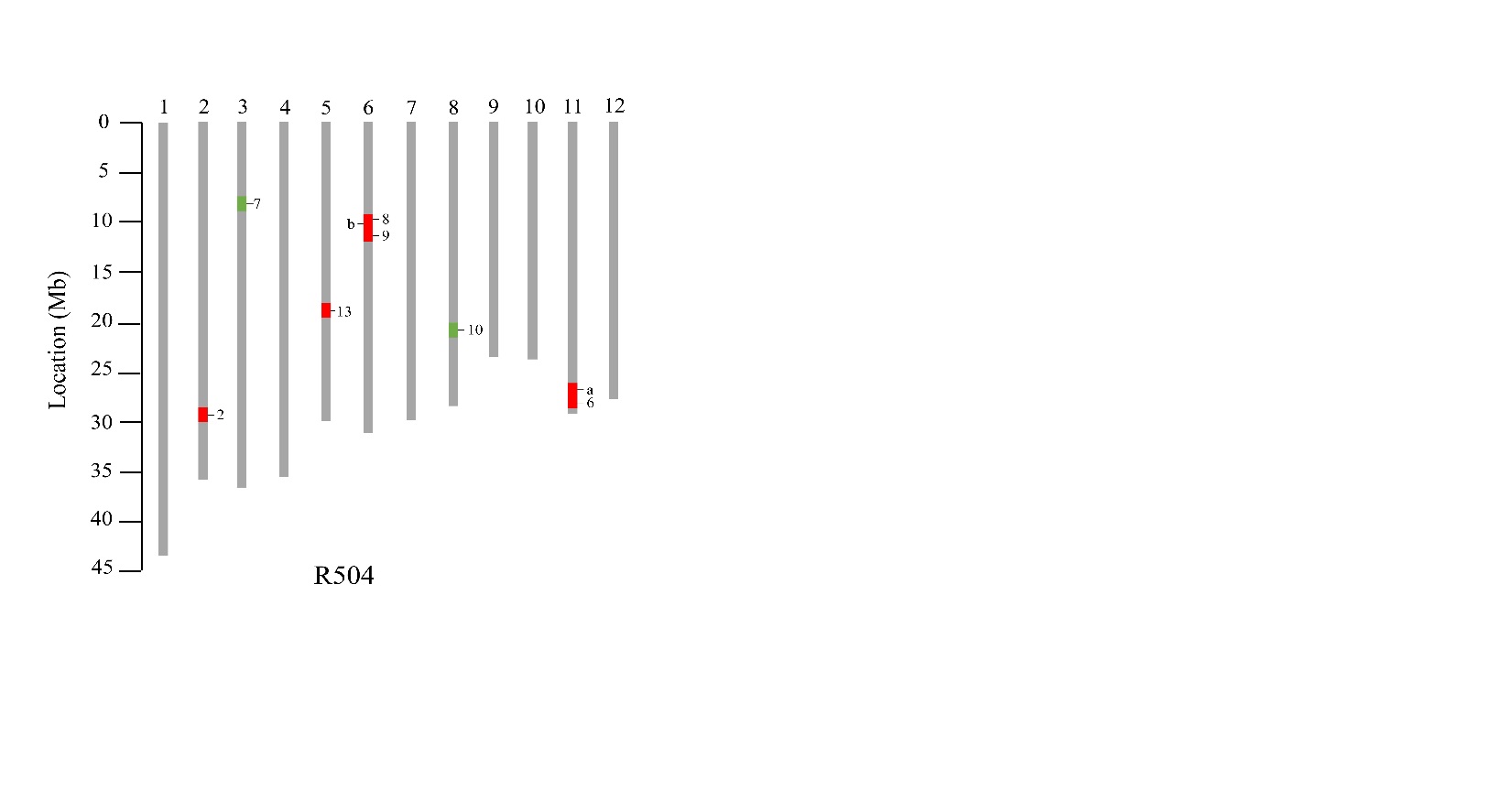


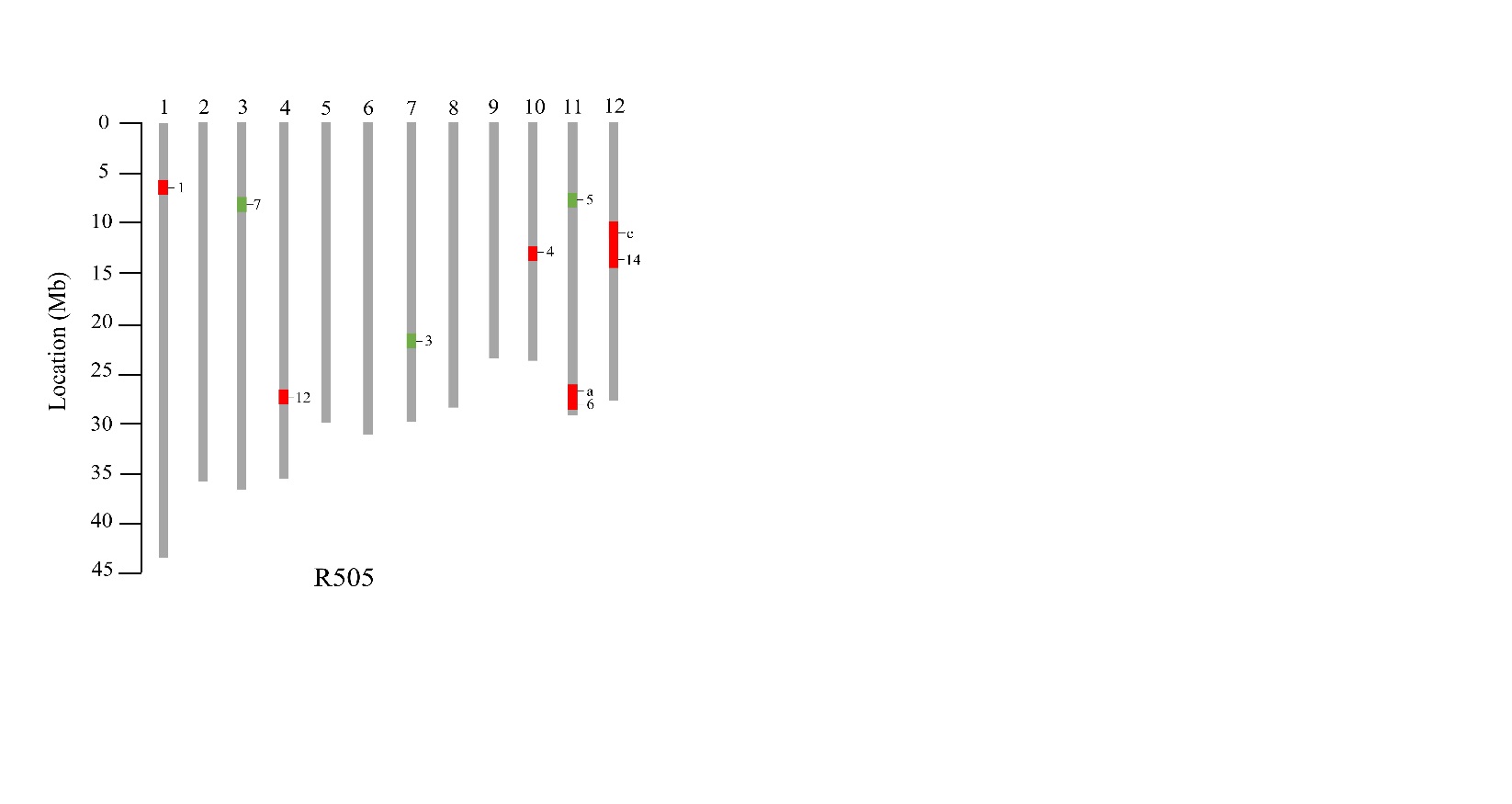

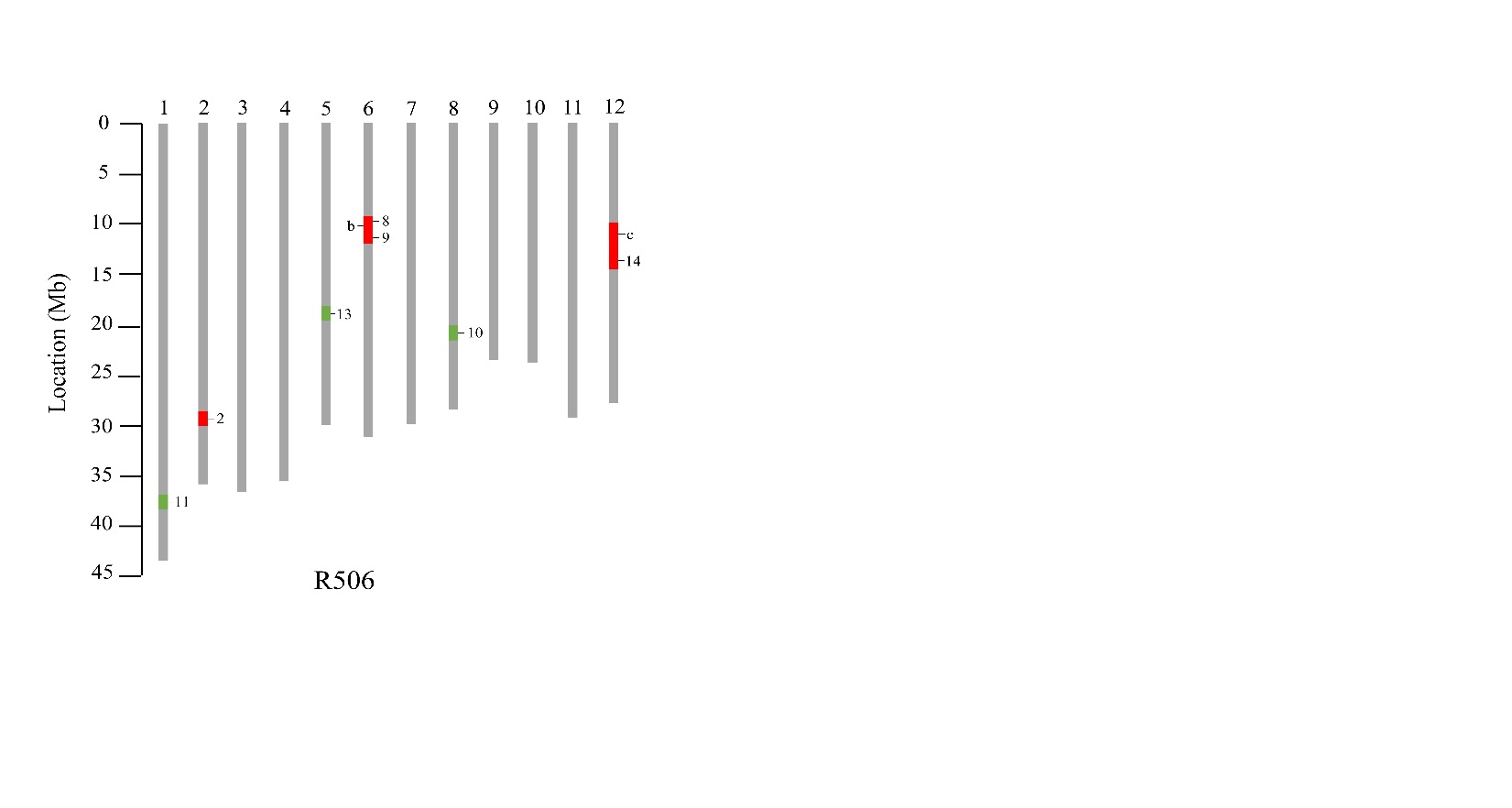


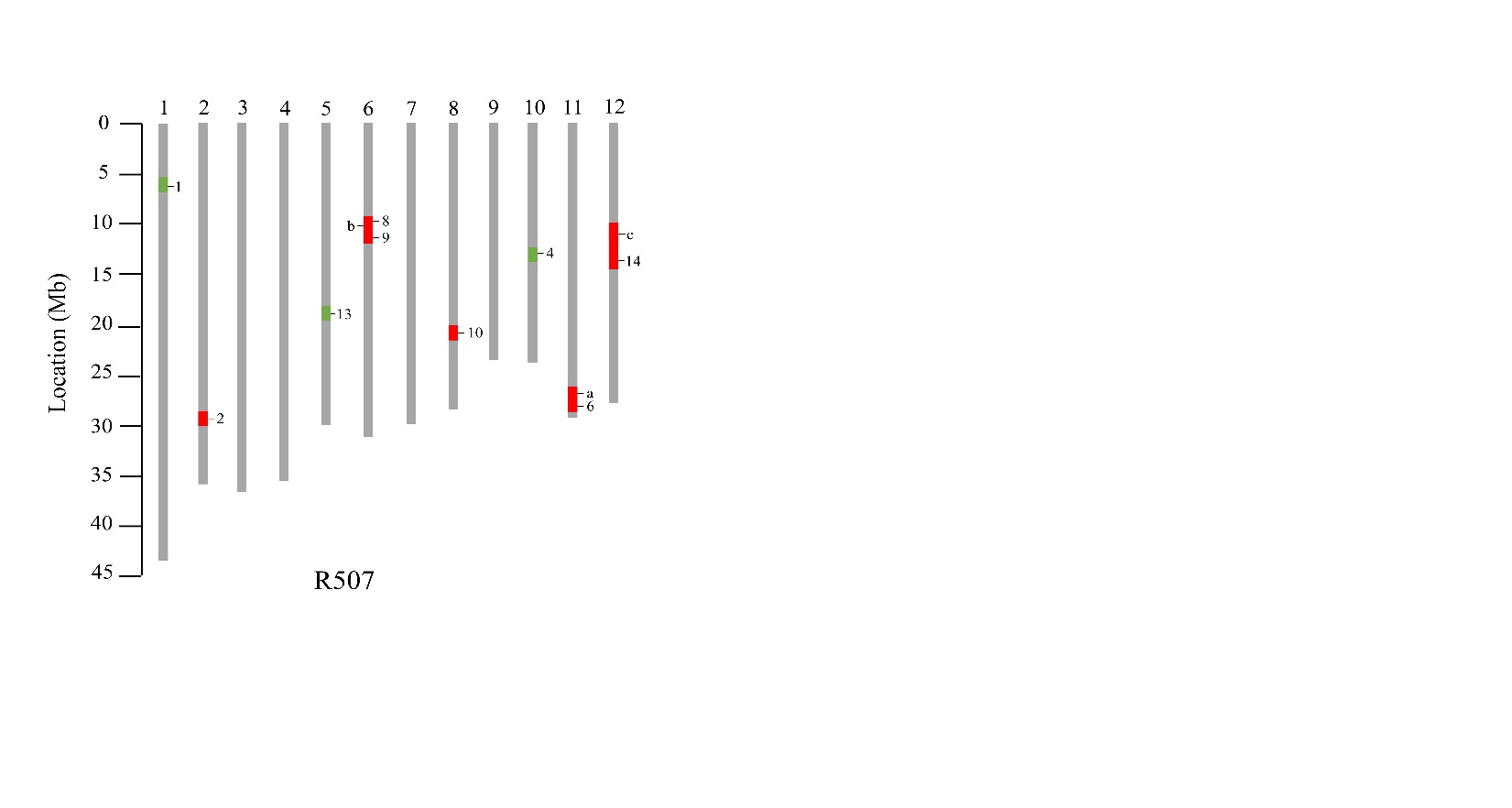


Fig. S1 Genetic background analysis of the seven improved lines by the remaining 14 SSR markers which detected polymorphic between the RP and the BC_3_F_1_ sample

Note: The blue lines indicate the chromosomal segments of the recurrent parent. The red and green lines indicate the chromosomal segments with homozygous and heterozygous genotypes where genomic fragments of the donor parents were introgressed. a, b and c means the three markers RM224, Ind306 and *Pita*-Ext/Int respectively, which were used for foreground selection. 1-14 represent the markers RM490, RM497, RM336, RM467, RM479, RM144, RM218, RM527, RM564 (RM19877), RM223, RM472, RM451, RM430 and RM179, respectively.
